# Lipid-mediated Protein-protein Interactions Modulate Respiration-driven ATP Synthesis

**DOI:** 10.1038/srep24113

**Published:** 2016-04-11

**Authors:** Tobias Nilsson, Camilla Rydström Lundin, Gustav Nordlund, Pia Ädelroth, Christoph von Ballmoos, Peter Brzezinski

**Affiliations:** 1 Department of Biochemistry and Biophysics, The Arrhenius Laboratories for Natural Sciences, Stockholm University, SE-106 91 Stockholm, Sweden; 2 Department of Chemistry and Biochemistry, University of Bern, Freiestrasse 3, 3012 Bern, Switzerland

## Abstract

Energy conversion in biological systems is underpinned by membrane-bound proton transporters that generate and maintain a proton electrochemical gradient across the membrane which used, e.g. for generation of ATP by the ATP synthase. Here, we have co-reconstituted the proton pump cytochrome *bo*_3_ (ubiquinol oxidase) together with ATP synthase in liposomes and studied the effect of changing the lipid composition on the ATP synthesis activity driven by proton pumping. We found that for 100 nm liposomes, containing 5 of each proteins, the ATP synthesis rates decreased significantly with increasing fractions of DOPA, DOPE, DOPG or cardiolipin added to liposomes made of DOPC; with e.g. 5% DOPG, we observed an almost 50% decrease in the ATP synthesis rate. However, upon increasing the average distance between the proton pumps and ATP synthases, the ATP synthesis rate dropped and the lipid dependence of this activity vanished. The data indicate that protons are transferred along the membrane, between cytochrome *bo*_3_ and the ATP synthase, but only at sufficiently high protein densities. We also argue that the local protein density may be modulated by lipid-dependent changes in interactions between the two proteins complexes, which points to a mechanism by which the cell may regulate the overall activity of the respiratory chain.

Energy conversion in biological systems involves a transmembrane proton electrochemical gradient that is maintained by membrane-bound proton transporters. The gradient is used, for example, for production of ATP by the F_1_F_o_ ATP synthase or for transmembrane transport (for review, see^1^). The enzymes that generate the transmembrane electrochemical gradient vary greatly between species. For example, mitochondria or aerobic bacteria employ a series of proton-transporting respiratory-chain complexes while phototrophic bacteria or plants use photosynthetic reaction centers to generate the transmembrane electrochemical gradient. Many of these primary proton pumps and transporters have been studied in detail. However, far less is known about their interactions within the membrane or the involvement of the membrane in the energy-conversion process. Of particular interest is the role of the membrane in functional interactions between membrane proteins that maintain the proton electrochemical gradient (e.g. proton pumps) and those that use the gradient (e.g. F_1_F_o_ ATP synthase). For example, membrane surface groups may be involved in lateral proton transfer that would compete kinetically with transfer via bulk water. This phenomenon has been addressed in the past in a number of experimental and theoretical studies^2^,^3^,^4^,^5^,^6^,^7^,^8^,^9^,^10^,^11^,^12^,^13^,^14^,^15^,^16^,^17^,^18^,^19^,^20^,^21^. For example, in a landmark study with purple membranes of *H. salinarium*, Heberle *et al*. found that protons released upon illumination of the light-driven pump bacteriorhodopsin are transferred significantly faster along the membrane surface than from the surface to bulk water^21^. Co-reconstitution of bacteriorhodopsin and ATP synthase in liposomes has been demonstrated in the past^22^,^23^,^24^,^25^,^26^.

In a recent study we described a system where the *bo*_3_ ubiquinol oxidase from *E. coli* was co-reconstituted with ATP synthase from the same bacterium^27^. The *bo*_3_ oxidase is a proton pump, which couples oxidation of membrane-soluble ubiquinol QH_2_ (Q_8_ in *E. coli*) to reduction of dioxygen to water^28^,^29^,^30^. The protons used for O_2_ reduction are taken up from the more negative (*n*) side of the membrane while the protons from QH_2_ oxidation are released to the more positive (*p*) side of the membrane. In addition, protons are pumped across the membrane, yielding a total stoichiometry that is equivalent to the transport of two positive charges from the *n* to the *p* side per electron transferred to O_2_. The combined system composed of the *bo*_3_ oxidase and ATP synthase in the same liposomes is thus a minimum model of oxidative phosphorylation of aerobic bacteria or mitochondria. In the present study, we used this model system to investigate the influence of the lipid composition on proton pumping coupled to ATP synthesis. We found that the ATP synthesis activity (rate) decreased dramatically upon addition of small amounts of negatively charged lipids such as DOPG, DOPA or cardiolipin, while the decrease in activity was less pronounced upon addition of the zwitterionic lipid DOPE. Furthermore, we found that this lipid effect on the coupled *bo*_3_-ATP synthesis activity is dependent on density of the two enzymes in the membrane.

## Results

### The effect of lipid composition on the coupled activity

**Figure 1:**
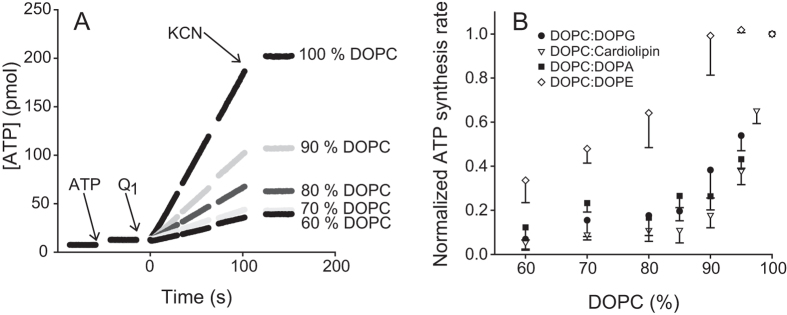
Coupled *bo*_3_-ATP synthase activity. (**A**) Changes in luminescence of the luciferin-luciferase couple as a result of ATP synthesis driven by an electrochemical gradient generated by the *bo*_3_ oxidase. The reaction was started at *t* = 0 upon addition of ubiquinol UQ_1_ (20 μM after mixing) to a tube containing liposomes with co-reconstituted ATP synthase and *bo*_3_ oxidase and DTT (2 mM). The reaction was stopped by addition of potassium cyanide (500 μM, at *t* ≅ 100 s), which inhibits the *bo*_3_ oxidase. At *t* = −50 s, a small amount (5 pmol) of ATP was added to normalize the signals. The traces shown are for 100 nm liposomes with DOPC fractions of (the remaining part is DOPG): 100, 90, 80, 70, 60% from the top to the bottom. The traces are divided in 30-s segments for technical reasons. Measurements were performed at ~22 °C in 20 mM Tris-PO_4_ at pH 7.5, 2.5 mM MgSO_4_, 80 μM ADP. (**B**) The ATP synthesis rates (slopes of the traces in A) measured (*n* is the number of measurements) with increasing amounts of DOPG (black circles, *n* = 9–12), DOPA (black squares, *n* = 5–7), DOPE (white diamonds, *n* = 2 (points at 5% and 15%), 7 (remaining points)) or cardiolipin (white triangles, *n* = 4 (point at 5%), 12 (remaining points)). The relative ATP synthesis rates are shown as a fraction of the activity in 100 nm liposomes with 100% DOPC (~90 ATP × s^−1^ × enzyme^−1^ for dialysis reconstitution).

In a first series of experiments, *bo*_3_ oxidase and ATP synthase were co-reconstituted in 100 nm DOPC liposomes, containing increasing relative amounts of DOPG, ranging from 0 to 40% DOPG. The protein concentration was adjusted to yield about five *bo*_3_ oxidases and five ATP synthases per liposome. Proton pumping of the *bo*_3_ oxidase was initiated by addition of DTT and ubiquinone UQ_1_ (Fig. 1A). Turnover of the *bo*_3_ oxidase results in formation and maintenance of a proton electrochemical gradient that is used by the F_1_F_O_ ATP synthase to convert ADP and inorganic phosphate to ATP. Production of ATP was monitored *in situ* using the luciferin/luciferase system (i.e. the luminescence was monitored). The ATP synthesis rate (referred to below as the coupled activity) was determined from the average of the slopes of the 2nd and 3rd segments after UQ_1_ addition (see Fig. 1A). At *t* ≅ 100 s the reaction was impaired by adding KCN, which blocks oxygen binding to the *bo*_3_ oxidase.

As seen in Fig. 1A, the coupled activity decreased, from that obtained with the 100% DOPC liposomes, with increasing DOPG content. Already at 5% DOPG, almost a 50% decrease in ATP synthesis rate was observed. (Fig. 1B). Less than 10% coupled activity was found in liposomes containing 40% DOPG. We also investigated the effect of addition of other lipids to the DOPC liposomes on the respiration-driven ATP synthesis (Fig. 1B). First, we added an increasing fraction of the negatively charged DOPA that lacks the head group of e.g. DOPC, rendering an additional ionizable group on the phosphate (p*K*_a_ ≅ 8). Similarly to DOPG, increasing amounts of DOPA led to a strongly decreased coupled activity, slightly more prominent than with DOPG. Next, we used cardiolipin as an additive to our DOPC liposomes and measured the coupled activity. The presence of cardiolipin severely decreased the coupled activity such that at 15% cardiolipin, less than 10% of the coupled activity was observed, compared to 100% DOPC liposomes. In order to investigate if this effect is restricted to additions of negatively charged lipids to the zwitterionic DOPC, we added increasing fractions of another zwitterionic lipid, DOPE. This lipid has a more conical shape and tends therefore to destabilize a flat membrane. However, it does not alter the overall surface charge. Although much less pronounced than for the negatively charged lipids, the presence of DOPE led also to a decreased coupled activity.

The size of the liposomes was only slightly affected by the different lipid compositions. When using a filter with a pore diameter of 100 nm for preparation of liposomes (used in the above-described measurements) we obtained a population with a mean diameter of 90 ± 25 nm with an asymmetric distribution (see Figure S1, supporting information). The mean diameter was independent of the lipid composition for DOPC:DOPG ratios of 100:0 or 60:40%, or DOPC:Cardiolipin 70:30%. However, the DOPC:DOPE vesicles were slightly larger, e.g. at a 70:30% mixture a mean diameter of 120 ± 15 nm was found. The size distribution did not change significantly upon protein reconstitution within the liposomes^27^. Below, we refer to the diameter of the liposomes by the size of the filter used to prepare the liposomes, even if the actual diameter is slightly different from that of the filter pore size (see Figure S1).

### The effect of lipid composition on the enzymatic activities

**Figure 2:**
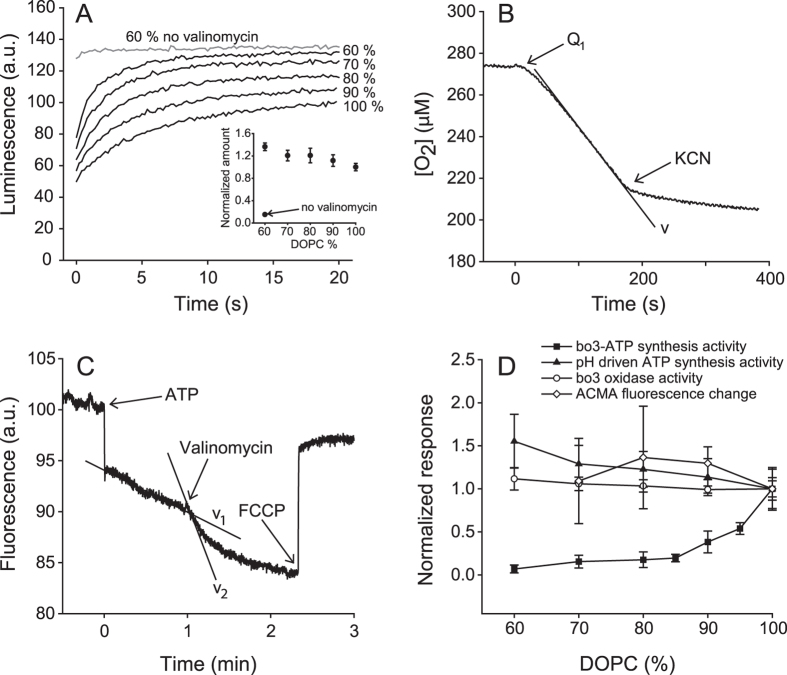
Individual activities of *bo*_*3*_ oxidase and ATP synthase. **(A)** Changes in the luminescence as a result of ATP synthesis. A transmembrane pH gradient was established as described in the Materials and Methods section (100 nm liposomes with ATP synthase, 60–100% DOPC (with DOPG). “a.u.” is “arbitrary units”. The inset shows the total amounts of ATP produced within the first 20 s (normalized to 100% DOPC, 145 pmol). Error bars: 4 measurements with two different samples. **(B)** Changes in oxygen concentration during turnover of the *bo*_3_ oxidase in 100 nm liposomes. The reaction was initiated at *t* = 0 by addition of ubiquinol UQ_1_ (20 μM) in the presence of 2 mM DTT (with valinomycin (10 μM) and FCCP (20 μM)) and stopped by addition of KCN (0.32 mM). The trace shown is for DOPC:DOPG = 80:20% (see panel **D** for data with other ratios). The rate was determined from the slope indicated as *v*. **(C)** Changes in fluorescence of (2.6 μM) ACMA (100% DOPC 100 nm liposomes with ATP synthase). Upon addition of 2.5 mM ATP (the rapid change is a mixing artifact) at *t* = 0 the fluorescence decreased with a slope, *v*_1_, which reflects the rate of proton pumping into the liposomes. Upon addition of 130 nM valinomycin, the acidification rate increased (*v*_2_). A “respiratory-control ratio” was calculated as *v*_2_/*v*_1_. (**D**) Summary of the data presented in panels (**A–C**). Within each data series, every point is normalized to that obtained with 100% DOPC (the remainder is DOPG). The (black squares) is the coupled *bo*_3_-ATP synthase activity shown for reference (taken from Fig. 1B). (black triangles) Initial slope (~3 s) of the luminescence change for each of the traces in panel (**A**). This slope reflects the maximal ATP synthesis, i.e. the activity of the ATP synthase (2 measurements). (white circles) Oxygen consumption activities of the *bo*_3_ oxidase (c.f. panel **B**) in the presence of uncouplers (3 measurements). (white diamonds) The ratio *v*_2_/*v*_1_ from panel **C**) (3 measurements).

Next, we investigated the effect of lipid composition on the activities of ATP synthase and *bo*_3_ oxidase alone. Because similar effects were observed upon addition of DOPG, DOPA or cardiolipin to the DOPC vesicles, in the following we focus on effects of addition of DOPG. To measure the ATP synthesis activity, the proteoliposomes were first incubated in a buffer with low pH and low potassium (in equilibrium with the inside) and then the bulk solution was rapidly diluted into a buffer with high pH, containing a higher potassium concentration and valinomycin (Fig. 2A). This procedure yields liposomes with a pH gradient (inside acidic) and a membrane potential (inside positive), the latter resulting from valinomycin-mediated diffusion of potassium into the liposomes (for a detailed procedure, see Materials and Methods section, and Figure S2). With increasing DOPG concentrations, the initial rates increased mildly, reaching a 1.6-fold increase with liposomes containing 40% DOPG (see panels **A** (traces) and **D** (rates) in Fig. 2), i.e. the lipid effect on the ATP synthase activity was opposite to that observed for the coupled activity. This result is in agreement with that from an earlier study^22^ showing that after build-up of an electrochemical proton gradient by bacteriorhodopsin, the ATP synthesis rate was a factor of ~2 higher upon addition of DOPA to DOPC liposomes.

We also determined the effect of lipid composition on the oxygen consumption rate of the *bo*_3_ oxidase after addition of quinol to proteoliposomes containing cytochrome *bo*_3_ (Fig. 2B). Within the tested range (0–40% DOPG), no influence of the lipid composition was observed on the steady-state activity of the *bo*_3_ oxidase (Fig. 2D).

Taken together, these data show that the activities of the two enzymes alone did not decrease with increasing fraction DOPG, which indicates that the decrease in the coupled *bo*_3_-ATP synthase activity was not due to a decrease in the activities of any of the two enzyme components.

### Influence of lipid composition on the proton permeability

Another explanation for the observed lipid effect could be an altered proton permeability with the different liposome preparations. A higher proton permeability with an increasing amount of DOPG would lead to a faster loss of the electrochemical proton gradient and consequently decreased ATP synthesis rates. Therefore, in the next series of experiments we estimated the leak rates for the different lipid compositions. First, we quantified the total amount of ATP formed upon establishment of a proton electrochemical gradient across a liposome membrane (Fig. 2A). The data show that the total change in luminescence (total amount of ATP formed) was similar, but slightly increasing with increasing DOPG content (Fig. 2A, inset), which indicates that over the time scale of the measurement (~20 s), the membrane did not significantly dissipate the proton gradient to different extents in the different lipid compositions. Furthermore, we also estimated the membrane proton permeability for the different lipid compositions by measuring the fluorescence of ACMA as a result of ATP driven proton translocation by the ATP synthase^31^,^32^ (Fig. 2C). ACMA fluorescence is quenched upon acidification of the inner volume, reflecting the proton-concentration difference across the membrane. Upon addition of ATP, the outwardly oriented F_1_F_o_ ATP synthases pump protons to the inside of the liposomes. Accordingly, we observed a decrease in fluorescence with a slope that is approximately proportional to the proton-transfer rate (the unresolved initial change in Fig. 2C is a mixing artifact). This rate increased upon addition of the uncoupler valinomycin, which dissipates the opposing electrical component of the electrochemical gradient formed by the ATP synthase. The ratio of the slopes after and before addition of valinomycin reflects the tightness of the membrane. The data for different DOPC:DOPG fractions are summarized in Fig. 2D and suggest that the leak rates were independent of the lipid composition.

The above-described ACMA-based method was used rather than measuring the respiratory-control ratio (RCR) of the *bo*_3_ oxidase because the *bo*_3_ oxidase has been shown to orient randomly in the membrane^33^,^34^ and the electron donor (ubiquinol) presumably does not discriminate between the two *bo*_3_ orientations. Therefore, the RCR value is small and does not reflect the true membrane tightness^34^. Nevertheless, we determined the RCR of the liposomes and found values in the same range (1.35 ± 0.15) for all lipid ratios (60–100% DOPC, with addition of DOPG).

Finally, the membrane proton permeability of membranes with the different lipid compositions were investigated by studying the changes in ACMA fluorescence upon establishment of a pH gradient across the liposome membrane with and without reconstituted ATP synthase and *bo*_3_ oxidase (see Figure S3, supporting information). No significant differences were observed over time scales of minutes between the different samples, which indicates that the observed decrease in the coupled *bo*_3_-ATP synthase activity with increasing DOPG content is not due to an increased proton leak.

### Effect of lipid composition on quinol diffusion within the membrane

**Figure 3:**
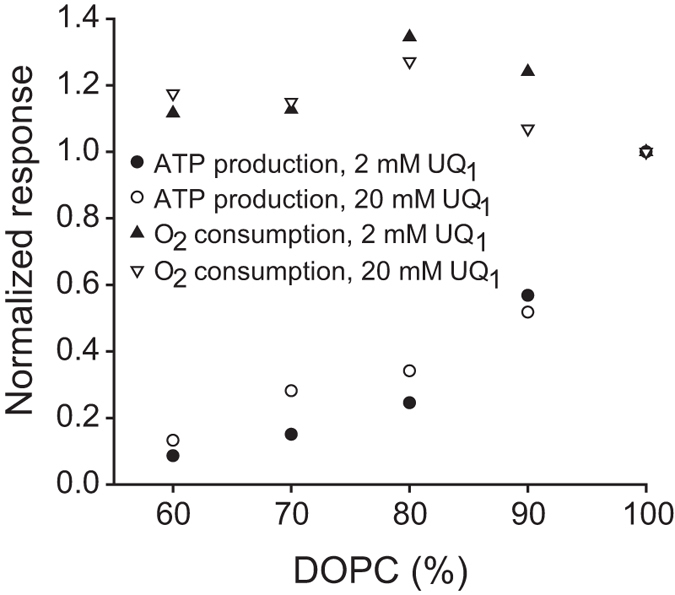
ATP synthesis and O_2_ consumption as a function of the fraction of DOPC for two different UQ_1_ concentrations. The remainder of the lipids is DOPG. The rates were measured with co-reconstituted *bo*_3_-ATP synthase in 100 nm liposomes for 2 μM and 20 μM UQ_1_, respectively (in the presence of 2 mM DTT). The activities were normalized to those obtained for 100% DOPC. Under these conditions, the ATP synthesis rates measured with 2 μM UQ_1_ were a factor of 3 smaller than those measured with 20 μM UQ_1_. The corresponding difference in the O_2_-consumption rates was a factor of 10.

Ubiquinone Q_1_ is a non-natural short chain length quinone that is soluble in aqueous solution at low concentrations. Unlike natural ubiquinone (Q_8_ in *E. coli* or Q_10_ in mitochondria), it can readily be reduced in aqueous solution by e.g. DTT. Because of its amphiphilic properties, it also partitions in the membrane, where it can reduce the *bo*_3_ oxidase. Consequently, UQ_1_ is commonly used as an electron mediator for different quinol-binding enzymes. To explore the possibility that loss of the coupled *bo*_3_-ATP synthesis activity with increasing DOPG content is due to altered membrane partition or slowed UQ_1_ diffusion within the membrane, we measured the activity at different UQ_1_ concentrations. Upon increasing the UQ_1_ concentration from 20 μM to 40 μM, the coupled activity with 100% DOPC liposomes increased by ≤10% and no further increase was observed upon increasing the UQ_1_ concentration to 100 μM. This control shows that at 20 μM UQ_1_ (the concentrations used in our experiments), electron transfer from UQ_1_ to the *bo*_3_ oxidase in the membrane was not rate limiting for the overall process. When lowering the UQ_1_ concentration to 2 μM, the cyt. *bo*_3_ activity decreased by a factor of 10, while the coupled activity decreased by a factor of ~3 (100% DOPC). We then measured the cytochrome *bo*_3_ and the coupled *bo*_3_-ATP synthase activities for liposomes consisting of different DOPC:DOPG ratios. As seen in Fig. 3, the activity of the *bo*_3_ oxidase was essentially independent of the lipid composition for both UQ_1_ concentrations (in the range 60–100% DOPC). The coupled *bo*_3_-ATP synthase activity decreased similarly with increasing fraction DOPG for both UQ_1_ concentrations, showing that the overall process of UQ_1_ diffusion, binding and electron transfer to the *bo*_3_ oxidase was not affected by the different lipid compositions.

### Effect of protein density on the coupled activity

**Figure 4:**
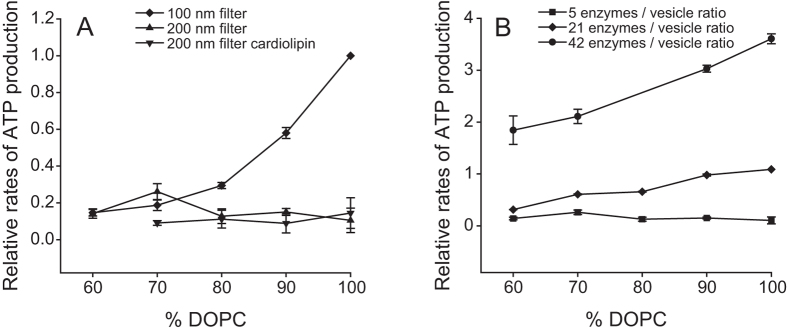
Coupled *bo*_3_-ATP synthase activity as a function of the protein density and vesicle size. (**A**) The coupled activity as a function of lipid composition for vesicles obtained with 100 nm (filled diamonds) and 200 nm (filled triangles) pore-size filters. The data is normalized to 100 nm vesicles containing 100% DOPC. The average number of proteins per vesicle was 5 cyt. *bo*_3_ and 5 ATP synthase. For the 100 nm vesicles, DOPC was mixed only with DOPG, while for the 200-nm vesicles DOPC was mixed with both DOPG (filled triangles up) and cardiolipin (filled triangles down). (**B**) The coupled activity as a function of lipid composition for vesicles obtained with 200 nm pore-size filters and with different number of proteins (as indicated) keeping the 1:1 ratio of *bo*_3_:ATP synthase. Data is normalized to 100% DOPC vesicles with 21 of each enzyme, which yields approximately the same lipid/enzyme ratio as for 5 of each enzyme in 100 nm vesicles (~2000 lipids/enzyme). The number of measurements was 2–4.

The data discussed above show that the catalytic activities of *bo*_3_ oxidase and ATP synthase did not decrease with increasing fractions of the lipids added to the DOPC liposomes. Consequently, the observed effects on the coupled activity are presumably attributed either to changes in the medium between the two proteins or interactions between the components. We thus also investigated the effect of the protein density (average distance between proteins) on the coupled activity. To investigate the coupled activities with a lower protein density, we prepared liposomes with about a factor of two larger diameter (using a 200-nm filter, yielding a mean diameter of ~170 nm, see Figure S1) keeping the protein concentrations the same, i.e. with an approximately four times smaller protein density. As seen in Fig. 4A, the dependence of the coupled activity on the lipid composition was lost with the larger liposomes. To test whether or not this absence of activity change upon altering the lipid composition was specific to DOPG, we also included cardiolipin in the DOPC liposomes and obtained the same results as with DOPG (Fig. 4A). To test whether or not the observed effect could be attributed to the larger liposomes rather than the smaller protein density, we also studied the coupled activity as a function of lipid composition for different protein-lipid ratios (while keeping the ratio *bo*_3_:ATP synthase at 1:1) in liposomes prepared with the 200-nm pore filter (Fig. 4B). As seen in the Figure, the dependence of the coupled activity on the lipid composition gradually appeared with the higher protein densities. However, the effect for the highest protein concentration was smaller than that observed for the liposomes prepared with the 100 nm pore size.

## Discussion

We have investigated the effect of lipid composition on rates of ATP synthesis catalyzed by ATP synthase, driven by an electrochemical proton gradient created and maintained by a co-reconstituted *bo*_3_ ubiquinol oxidase. Addition of small amounts of DOPG, DOPA or cardiolipin (negatively charged head groups) to the DOPC membranes resulted in a drop in the activity (see Fig. 1). When a gradually increasing fraction of the zwitterionic DOPE was added to the DOPC liposomes we also observed a drop in the activity, but not as dramatic as with the negatively charged lipids. Before we discuss possible explanations of the observed effect at a molecular level, we first argue that a number of trivial lipid-dependent effects (*i*-*iv*, listed below) could be excluded:

*(i)* The activities of the two enzymes themselves did not decrease upon addition of the other lipid components to the DOPC-containing liposomes (summarized in Fig. 2D). The increase in the activity of the ATP synthase with increasing fraction DOPG (by a factor of ~1.6 at 40% DOPG) is qualitatively consistent with earlier results from studies of the effect of addition of DOPA to DOPC liposomes^22^. The activity of cytochrome *c* oxidase is dependent on lipid composition, but primarily when changing the hydrocarbon chain length^35^ (for review see also^36^). In a recent study we found that the rate of specific proton-coupled electron transfer steps in cytochrome *c* oxidase are dependent on the presence of a surrounding membrane (as compared to detergent-solubilized oxidase), however, the reaction steps that are most strongly affected by changing the lipid composition (15,16, e.g. proton uptake during reduction and the so-called **P** → **F** reaction, τ ≅ 100 μs^37^), are not rate-limiting for the overall turnover for the membrane-reconstituted oxidase.

*(ii)* The diffusion of the quinol electron donor was lipid independent (Fig. 3).

*(iii)* We also argue that the reconstitution efficiency was lipid independent. First, the activity of the reconstituted *bo*_3_ oxidase or ATP synthase *alone* were independent of lipid composition (point (*i*)). Second, the liposome size and thereby the concentration was lipid independent upon addition of DOPG, DOPA or cardiolipin to the DOPC liposomes, which means that the activity per liposome was lipid independent suggesting a lipid-independent reconstitution efficiency.

*(iv)* Lipid-dependent proton leaks across the membrane were excluded based on a number of independent observations: (*a*) the total amount of ATP formed upon establishment of a proton-concentration gradient was independent of lipid composition (Fig. 2A,D, (*b*) the ratio of the rates of ACMA-fluorescence changes with/without valinomycin, as a result of formation of an ATP driven proton gradient, was independent of lipid composition (Fig. 2C,D), (*c*) the decay rate of an artificial proton gradient, formed by changing the pH on the outside of the liposomes, was independent of lipid composition (Figure S3). The proton leak rate can also be determined by measuring the respiratory control ratio (RCR) for the *bo*_3_ oxidase. As already mentioned above, this ratio is defined as the oxidase activity in liposomes in the absence (coupled liposomes) and presence of ionophores (uncoupled liposomes), i.e. the RCR is a measure of how tight the membrane is to proton leaks. Even though the obtained RCR values were small for reasons outlined in the Results section, they were relatively constant for the different lipid compositions, which is also consistent with an unchanged leak rate. We also note that results from earlier studies show that the proton permeability coefficients may vary by several orders of magnitude depending on the lipid composition, especially between natural sources and synthetic lipids (summarized in^38^). However, the experimental conditions in our study differ from those of the studies summarized in^38^. Furthermore, in general, the reported proton conductance (leakiness) across native mitochondrial membranes is much larger than that of artificial membranes^38^. Because this proton conductance apparently does not interfere with ATP production in the native membranes, the smaller proton leaks measured across artificial membranes are presumably not relevant when measuring ATP synthesis driven by charge separation and proton pumping.

Another point that must be considered is the orientation of the enzymes in the membrane and the fraction of the liposome population in which ATP synthesis occurs. Data from earlier studies have shown that the ATP synthase exclusively orients with the F_1_ headpiece to the outside^39^. In other words, electrochemically-driven ATP synthesis requires the membrane potential to be positive inside the liposomes (i.e. promoting a proton flux from the inside to the outside). Even though the *bo*_3_ oxidase orients randomly in the membrane, our data show that in a significant fraction of the liposomes the majority of the *bo*_3_ oxidases are oriented such that the net proton flux is from the outside to the inside of the liposomes (because we observe synthesis of ATP). The question then arises, how large is this fraction of liposomes? The ATP synthesis rate of the investigated system was up to 90 ATP s^−1^ enzyme^−1^, which is similar to rates reported earlier for other *in vitro* systems^27^,^40^. Because the activity is given per ATP synthase molecule, this observation indicates that a majority of the ATP synthases (i.e. a majority of the liposomes) are involved in the studied reaction. A related question is, whether or not the orientation of *bo*_3_ oxidase changes with an altered lipid composition. The relatively constant RCR values indicate a lipid-independent orientation of the *bo*_3_ oxidase, but this is a weak argument given the small RCR values. A stronger argument for an unaltered *bo*_3_ oxidase orientation as a function of lipid composition is obtained from the data in Fig. 4A. These data show a lipid-independent coupled activity for the 200-nm liposomes, which indicates that the relative orientation of the ATP synthase and *bo*_3_ oxidase is preserved. Because the 100 nm liposomes are prepared using the same method, only using a filter with smaller apertures, these data indicate that the observed lipid dependence in the coupled activity is not due to changes in orientation of the enzyme. This conclusion is also supported by the data in Fig. 4B, which show a gradual increase in the lipid dependence of the coupled activity with increasing protein density in the membrane.

The overall coupled *bo*_3_-ATP synthase reaction, as studied here, can roughly be divided into three steps: proton pumping by the *bo*_3_ oxidase, proton transfer from the oxidase to the ATP synthase and ATP synthesis by the F_1_F_o_ ATP synthase. The data show that neither the activity of the *bo*_3_-oxidase nor that of the ATP synthase is sensitive to the membrane lipid composition (with the lipid combinations tested here). The effect of lipid composition is only observed on the coupled activity (see Fig. 1), which suggests that the lipid composition influences proton exchange between the two proteins. The data in Fig. 4A show that the coupled activity at 100% DOPC decreased by about a factor of 10 upon increasing the diameter of the liposomes by a factor of ~2 (i.e. the surface area by a factor of ~4), which suggests that the coupled activity is strongly dependent on the distance between proton donor and acceptor enzymes. The observation that the lipid composition influences the coupled activity and that this activity is dependent on the average distance between the *bo*_3_ oxidase and the ATP synthase suggests that the proton exchange between the two proteins does not occur exclusively via bulk solution, but involves the membrane. This conclusion is also supported by the observation that the lipid influence was lost in the larger liposomes, indicating that the membrane influences the coupled activity only when the distance between the oxidase and the ATP synthase is below a certain threshold (the weaker dependence of the coupled activity observed with the DOPE liposomes, Fig. 1, is also consistent with the slightly larger size of these liposomes, see Figure S1). The lipid effect could be partly recovered in the larger liposomes by increasing the protein density (Fig. 4B). In other words, below a certain protein density threshold, proton exchange presumably occurs via the bulk solution yielding a lipid-independent coupled activity. Another support for this scenario is offered by our recent finding that ATP synthesis during proton pumping by the *bo*_3_ oxidase is observed also in the presence of small concentrations of uncouplers^27^, a phenomenon reported earlier in the literature and referred to as “mild uncoupling”^41^. We note that the system investigated here is not at equilibrium because protons are continuously pumped by the oxidase. Consequently, the phenomenon earlier termed “mild uncoupling”^41^ could be explained by a local surface-to-surface proton gradient during proton pumping even in the presence of an ionophore.

**Figure 5:**
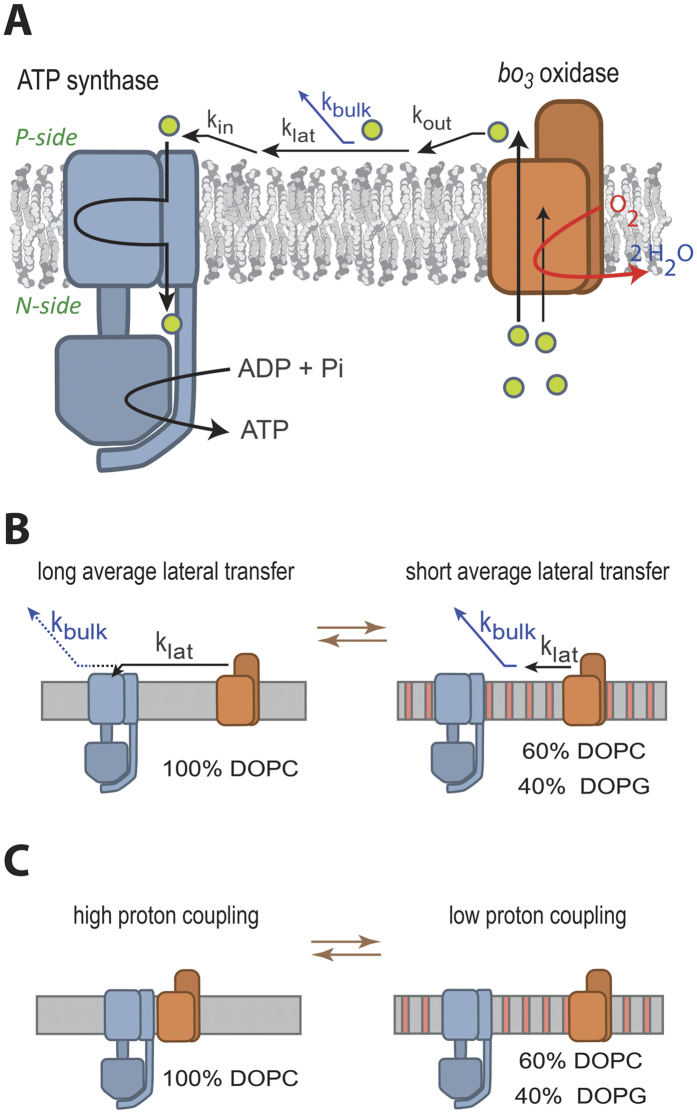
Schematic model illustrating the conclusions. See text for explanation.

As briefly outlined in the Introduction, it has been proposed that protons may be transferred laterally, along the membrane surface from a donor to an acceptor, if the lateral proton transfer is faster than proton equilibration with the bulk solution. Assuming lateral proton transfer, the proton transfer on the *p*-side surfaces can be divided into three main steps (Fig. 5A): (*i*) the proton would originate from a protonatable group located at the end of a proton-exit pathway of the *bo*_3_ oxidase from where it would be transferred, possibly *via* other protonatable surface groups on the enzyme, to the membrane (*k*_out_ in Fig. 5A), (*ii*) lateral proton transfer along the membrane surface (*k*_lat_), (*iii*) proton transfer from the surface, possibly *via* other protonatable surface groups on the ATP synthase, to a protonatable group at the entry point of a proton pathway (*k*_in_). The same sequence of events would take place at the *n*-side surface where the ATP synthase is the proton donor and the *bo*_3_ oxidase is the acceptor (not shown in Fig. 5).

Because we find it unlikely that proton exchange between the proteins and the membrane (c.f. rate constants *k*_in_ and *k*_out_) is influenced by the protein density (the proteins occupy less than 10% of the surface area), the data in Fig. 4 suggest that rather proton transfer along the surface (c.f. rate constant *k*_lat_) is responsible for the lipid effect on coupled activity. Assuming a mechanism where proton transfer between the two proteins occurs via the membrane surface, the observed effect of lipid composition on coupled activity could be explained either by (*i*) lipid-dependent changes in the distance/rate of lateral proton transfer (i.e. how far the proton is transferred along the surface before dissipating into solution) or by (*ii*) lipid-dependent changes in the average distance between *bo*_3_ oxidase and ATP synthase in the membrane. At present we can not discriminate between these two scenarios, but briefly discuss a number of relevant issues related to these mechanisms. Point (*i*) has been addressed extensively in the literature (see references in the Introduction section). Lateral proton transfer involves multiple proton transfers between surface groups with overlapping Coulomb cages that extend over distances on the order of ~1 nm (reviewed in^9^,^42^). If the transfer involves multiple hops, e.g. between lipid head groups, the proton may be transferred over longer distances, where the largest values extend beyond the size of the vesicles used in this study^43^. In other words, lateral proton transfer is possible. Next, we discuss the effect of changes in lipid head group composition on lateral proton transfer. About the same effect on the coupled *bo*_3_-ATP synthase activity was observed for all negatively charged lipids, DOPG, DOPA or cardiolipin. Results from earlier kinetic^42^,^44^ and equilibrium^12^,^14^ studies have shown that an increased fraction of negatively charged groups accelerates protonation of a site at the membrane. These earlier observations qualitatively contradict the findings from the present study. Furthermore, the coupled *bo*_3_-ATP synthase activity decreased also upon addition of DOPE to the DOPC membrane, which indicates that charge alone is not responsible for the observed effect as both head groups are zwitterionic. On the other hand, the decrease in the coupled activity was much less pronounced with DOPE than for the negatively charged head groups. As noted in^45^, for the PC and PE head groups, the relative positions of the positive and negative charges differ. These differences in the geometrical arrangement could yield a scenario, where with DOPE the membrane proton acceptor or donor displays a behavior that, at least to some extent, resembles that observed for the negatively charged lipids. Furthermore, as already noted above, DOPE has a more conical shape than DOPC and tends therefore to destabilize a flat membrane, which may influence proton diffusion along the surface. On the other hand, Springer *et al*.^46^ suggested that protons migrate along membrane surfaces not via surface groups but through an interfacial water layer and that this proton diffusion is independent of the lipid composition (but, see^8^). Independently of the mechanism of surface proton diffusion, the trajectory of proton transfer between two surface sites (e.g. a proton donor and acceptor site) is dependent on the relative rates of proton transfer along the surface and proton escape to the bulk solution (as well as diffusion in the bulk phase). Even if the proton diffusion rate along the membrane would be independent of the head group composition, the probability of proton escape to the bulk solution could be lipid dependent. Assuming that the average distance that a proton is transferred along the surface before its escape to the bulk water decreases with increasing fraction negative lipids (or DOPE), the ATP synthesis rate would decrease (see Figs 1B and 4). This is because for a given average density of cytochromes *bo*_3_ and ATP synthases, the probability that a proton migrates from the *bo*_3_ oxidase to the ATP synthase along the surface decreases when the average distance of proton transfer along the surface decreases. Consequently, a progressively larger fraction of protons is transferred via the bulk solution. At 60% DOPC with 100 nm liposomes, the ATP synthesis rate saturates at the lowest level (see Fig. 4), which according to the model would mean that under these conditions all protons escape into the bulk solution. This scenario (model) is supported by the observation that the rate at the saturation level (measured at 60% DOPC) is about the same as the (lipid-independent) rate measured with 200-nm liposomes (see Fig. 4). In the latter case, the lipid-independent rate of ATP production is assumed to indicate that proton transfer occurs only via the bulk solution.

As outlined in point (*ii*) above, an alternative explanation for the lipid effect on the coupled activity is that the average equilibrium distance between the *bo*_3_ oxidase and ATP synthase is dependent on lipid composition, but only if the membrane protein density is increased above a certain threshold. Above this threshold direct interactions between *bo*_3_ oxidase and ATP synthase would be possible, but vary with lipid composition. According to this scenario, the mechanism of proton transfer between the *bo*_3_ oxidase and ATP synthase would involve the membrane surface, but the rate of this transfer would not change with lipid composition (or this process is never rate-limiting). Instead, the main effect of the lipid composition would originate from changes in the average distance between the *bo*_3_ oxidase and ATP synthase. Relatively large patches composed of the complexes involved in oxidative phosphorylation were observed in the *E. coli* membrane^47^ indicating a non-random distribution of these complexes. Even though during the time of proton transfer between *bo*_3_ and ATP synthase the two proteins may diffuse a significant distance (compared to the size of liposome) (see e.g.^48^), the probability of close encounter of the two proteins could be lipid dependent. Of course, we can not exclude a combined effect of (*i*) and (*ii*).

### Summary

Our data show that the coupled activity may be decreased by a factor of 10 (from that observed with pure DOPC liposomes) by changing the lipid composition. We argue that the effect is attributed to proton exchange between the proton pump (*bo*_3_ oxidase) and the ATP synthase along the membrane surface. Our data can not uniquely discriminate between lipid-dependent changes in rates of lateral proton transfer or lipid-dependent changes in distance between the *bo*_3_ oxidase and ATP synthase. In any case, the observations point to a mechanism by which the living cell may regulate ATP synthesis by modulating interactions between proton pumps and the ATP synthase rather than by regulating the activity of each component separately. In addition to being regulated by the lipid composition, such interactions may be regulated through up- and down-regulation of small proteins that mediate more specific interactions between the components of the respiratory chain^49^.

## Materials and Methods

### Enzyme expression and purification

F_1_F_O_ ATP synthase was expressed from plasmid pBWU13-βHis in *E. coli* strain DK8 and purified as described^39^. Quinol-type *bo*_*3*_ oxidase was expressed from plasmid pETcyo in *E. coli* strain C43 and purified as described (see^50^ also^51^).

### Liposome preparation

Liposomes were prepared from 1,2-dioleoyl-*sn*-glycero-3-phosphocholine (DOPC), 1,2-dioleoyl-sn-glycero-3-phosphate (DOPA), 1,2-dioleoyl-sn-glycero-3-phosphoethanolamine (DOPE), 1,2-dioleoyl-sn-glycero-3-phospho-(1′-rac-glycerol) (DOPG) and 1′,3′-bis[1,2-dioleoyl-sn-glycero-3-phospho]-sn-glycerol (TO-cardiolipin), all purchased from Avanti Polar Lipids Inc. The lipids were stored in chloroform at −20 °C until use. The lipid stock solutions were mixed at different ratios (see Figure legends) and the chloroform was evaporated under nitrogen followed by vacuum evaporation. The lipid mixture was re-suspended at a 10 mg/ml lipid concentration in a buffer containing 20 mM HEPES pH 7.5, 2.5 mM MgSO_4_, 50 g/l sucrose and subjected to at least 6 freeze-thaw cycles (1 min in liquid nitrogen, then at 30 °C until thawed followed by 30 s vortexing). Finally, liposomes were formed by extrusion (>20 times) of the mixture through 100 nm or 200 nm Nuclepore membranes (Whatman Ltd).

### Enzyme reconstitution

A solution of 0.70 μM F_1_F_o_ ATP synthase and 0.70 μM *bo*_*3*_ oxidase was mixed with 0.14 μM liposomes in the presence of 0.4% sodium cholate, yielding a theoretical protein-per-liposome ratio of ~5 for each enzyme (see “supplementary information”). The detergent was removed using a pre-packed gel filtration column (PD-10, GE healthcare). Alternatively, to keep the sample volume at a minimum, we designed a simple dialysis method, which allowed us to dialyze 100 μl volumes. The dialysis was performed in the caps of standard 1.5 ml Eppendorf tubes. Dialysis membranes (8 kDa, Millipore) were stretched across the cap and the cap was plugged into the tube to secure the membrane. About 80% of the tube was cut off, which allowed access of the dialysis buffer to the membrane. The samples were then dialyzed over night at 4 °C against 100 ml dialysis buffer (20 mM Hepes, pH 7.5, 2.5 mM MgSO_4_, 50 g/l sucrose). For samples used for ACMA and oxygen-consumption measurements (see below), 100 mM KCl was included in the dialysis buffer.

### Respiration-driven ATP synthesis measurements

Measurements of respiration-driven ATP synthesis were performed as described^27^. To a solution containing 480 μl of measuring buffer (20 mM Tris-PO_4_, pH 7.5, 10 μl of luciferin/luciferase (CLSII, Roche, prepared according to the manufactures protocol), 2 μl ADP (20 mM) and 1 μl DTT (1 M), 10 μl liposomes was added and a baseline was recorded. Next, 5μl ATP (25 μM) was added at the beginning of each measurement to normalize the signal against a defined ATP amount. The reaction was then started by addition of 1 μl of ubiquinol Q_1_ (10 mM) and ATP synthesis was followed for at least 90 s.

### Measurements of the ATP synthase activity

ATP synthesis driven by the combination of an acid-bath treatment and a potassium/valinomycin diffusion potential was performed as described^52^. First, a sample of 20 μl proteoliposomes was mixed with 20 μl acidification buffer (0.2 M MES-NaOH, pH 5.5, 1 mM KCl) and incubated for 10 min at room temperature to allow equilibration of the inner and outer proton concentration. The liposome sample was then supplemented with 2 μl valinomycin (20 μM stock solution) and placed into the luminometer (10 points per second). ATP synthesis was initiated by rapid addition of 480 μl measuring buffer containing 20 mM Tris-PO_4_, pH 8, 100 mM KCl, 2.5 mM MgSO_4_, 10 μl of luciferin/luciferase (HS, Roche, prepared according to the manufactures protocol) and 2 μl ADP (20 mM). Mixing time of the two solutions was estimated to be ~1 s. After ATP synthesis had ceased, 5 μl ATP (25 μM) was added to calibrate the measured changes in light intensity. The initial (maximal) activity was estimated using the initial slope of the trace (first 3 seconds).

### bo_3_ oxidase activity measurements

The *bo*_3_ oxidase steady state activity was determined by measuring oxygen consumption using an oxygraph equipped with a Clark-type electrode (Hansatech). To a solution containing 970 μl buffer (20 mM Tris-HCl pH 7.5, 2.5 mM MgSO_4_, 100 mM KCl), 4 μl DTT (1 M), 5 μl valinomycin (2 mM) and 5 μl FCCP (5 mM), 20 μl liposomes was added and a baseline recorded. The reaction was initiated upon addition of 2 μl ubiquinone Q_1_ (10 μM) and turnover was recorded for several minutes. In control experiments, the reaction was stopped by addition of 8 μl KCN (40 mM) blocking oxygen binding to the *bo*_3_ oxidase.

### ATP hydrolysis and membrane tightness measurements

ATP dependent H^+^-transport into proteoliposomes by reconstituted *E. coli* ATP synthase was measured as described^53^. Briefly, to 1.5 ml of measurement buffer (10 mM Hepes-NaOH, pH 7.5, 2.5 mM MgSO_4_, 100 mM KCl), 2 μl 9-Amino-6-Chloro-2-Methoxyacridine (ACMA) (2 mM) and 30 μl liposomes was added and a baseline recorded. Proton-pumping was initiated by the addition of 5 μl ATP (200 mM) and recorded as a decrease in fluorescence. After 30 s, 1 μl valinomycin (100 μM) was added to dissipate the opposing membrane potential. The ratio of the slopes in fluorescence decreases before and after addition valinomycin addition was used to estimate the tightness of the membrane. Fluorescence signals were measured on a Cary Eclipse (Varian) and excitation and emission wavelengths were set at 410 nm and 480 nm, respectively.

## Additional Information

**How to cite this article**: Nilsson, T. *et al*. Lipid-mediated Protein-protein Interactions Modulate Respiration-driven ATP Synthesis. *Sci. Rep.* **6**, 24113; doi: 10.1038/srep24113 (2016).

## Supplementary Material

Supplementary Information
