# Supplementary material for: Lipid-mediated Protein-protein Interactions Modulate Respiration-driven ATP Synthesis
Source: Sci Rep. 2016 Apr 11;6:24113. doi: 10.1038/srep24113 (PMC4827085; doi:10.1038/srep24113)
Supplement: Supplementary Information [file srep24113-s1.doc]

Supporting Information

Lipid-mediated Protein-protein Interactions Modulate Respiration-driven ATP Synthesis

Tobias Nilsson^1^, Camilla Rydström Lundin^1^, Gustav Nordlund^1^, Pia Ädelroth^1^, Christoph von Ballmoos^1,2^*, Peter Brzezinski^1^*

^1^ Department of Biochemistry and Biophysics, The Arrhenius Laboratories for Natural Sciences, Stockholm University, SE-106 91 Stockholm, Sweden.
^2^ Department of Chemistry and Biochemistry, University of Bern, Freiestrasse 3, 3012 Bern, Switzerland

## Estimation of the liposome concentration and the number of enzymes per liposome

The number of lipids per liposome is estimated as outlined below. The total area of the inner and outer leaflets of a spherical liposome is:

$$A=4\pi\frac{\left(\frac{d}{2}\right)^{2}+ \left(\frac{d}{2}-l\right)^{2}}{A_{\text{DOPC}}}$$

where *d* is the diameter of the liposome (outer surface), *l* is the thickness of the bilayer (assumed to be 5 nm) and *A*_DOPC_ is the lipid surface area, which for DOPC is 70Å^2^ {Nagle, 2000 #3808}. Using these parameters, the number of lipids in a 100-nm liposome is estimated to be ~8·10^4^. The molecular weight of DOPC is 786 g/mol, which for a 10 mg/ml lipid concentration (used in the present study) gives a liposome concentration of ~0.16 μM. During reconstitution the liposome solution was slightly diluted to yield a liposome concentration of ~0.14 μM. To obtain a final concentration of five of each enzyme per liposome, we added ~0.7 μM of both *bo_3_* oxidase and ATP synthase during reconstitution. The values were adjusted to account for differences in the surface area of each of the lipids used in this study.

## Liposome size and concentration determination

Liposomes of uniform size were formed by extrusion of a liposome suspension through filters with diameters of 100 nm or 200 nm. Samples were diluted in phosphate buffered saline (PBS) (15 mM NaHPO_4_; 2.7 mM KH_2_PO_4_; 205 mM NaCl; 4 mM KCl) by factors of 10, 100 or 1000 and analyzed using Tunable Resistive Pulse Sensing (TRPS) (IZON Science qNano system). Calibrations were made with 100 nm and 200 nm monodisperse, carboxylated polystyrene particles for size and concentration measurements (CPC100B and CPC200B calibration particles from IZON Science). The data are presented in **Figure S1**.

## Changes in the luminescence of the luciferase-luciferin couple as a result of ATP synthesis driven by an electrochemical gradient

The experiment is described in the Materials and Methods section and illustrated in **Figure S2**. The acidified liposomes (50 μl) were supplied with 400 nM valinomycin in an Eppendorf tube and placed in the luminometer, and the measurement was started. However, this gives only a very small signal due to the absence of the luciferase system. During measurement, the liposomes were then mixed by injection of 450 μl measuring buffer (high pH, high potassium, ADP, phosphate and luciferase/luciferin) with a syringe. Mixing lasted ~1 s and we measured for another 25 s. After that, 5 μl of 25 μM ATP solution was added and the luminescence was measured again. The luminescence increase upon this addition was then used to normalize every trace individually.

## Proton leak determination using ACMA

Liposomes with a diameter of 100 nm were prepared as described in Materials and Methods, but in a buffer of 10 mM MES/MOPS pH 7.5 (instead of 20 mM HEPES buffer). A volume of 100 μl of these liposomes was mixed with 100 μl of a solution composed of 100 mM MES at pH 6 and the liposomes were incubated for at least 4 hours at 4°C to reach complete pH equilibration. A volume of 50 µl of these liposomes was then added to 1.5 ml buffer solution with pH 8 (20 mM Hepes, 50 mM K_2_SO_4_, 650 nM valinomycin and 1.3 nM ACMA) in a 1 ml cuvette. Fluorescence was monitored at 480 nm emission, with 410 nm as excitation wavelength (Cary Eclipse Fluorometer) for various time intervals. Nigericin (10 μM final concentration) was then added to remove the proton gradient for normalization purposes. The data are summarized in **Figure S3A**. No differences in the leak rates were observed for liposomes composed of 100 % DOPC and those composed of 60:40 % DOPC:DOPG.

## Proton leak determination with Pyranine

The pH sensitive ratiometric fluorescent dye pyranine was incorporated into empty liposomes or liposomes containing ATP synthase (270 μM final concentration added before freeze-thawing). The liposomes were composed of lipids with different DOPC:DOPG ratios. Pyranine on the outside of the liposomes was removed and the buffer was exchanged to 2 mM MES/MOPS pH 7.5, 5 mM MgCl_2_ on a PD10 desalting column. Samples were diluted 1:10 in the same buffer and incubated at 4°C overnight. To change the pH of the bulk solution from 7.5 to 6.5, HCl was added (5 μl, 0.5 M) and the change in pyranine fluorescence from the inside of the liposomes was monitored over time at 510 nm when exciting the dye at 406 nm and 460 nm, respectively (**Figure S3B**). The intensity ratio of emitted light at 406 nm and 460 nm was then used to determine the pH value. After ~3.5 min, 10 mM (final concentration) of the proton ionophore NH_4_Cl was added for normalization purposes. The experiment was performed in the presence of the quencher *p*-xylene-bis-pyridinium (DPX) to suppress fluorescence signal from remaining pyranine on the outside of the liposomes.

# Supplementary Figures and Legends

Figure S1. The liposome size distribution.

The particle size and concentration were measured using Tunable Resistive Pulse Sensing (TRPS). Calibration was made with 100 nm and 200 nm monodisperse, carboxylated polystyrene particles. **(A)** Liposomes prepared using the 200-nm filter. The measurements were done for 60, 80 and 100 % DOPC (the remaining part was DOPG). The mean diameter was 170±25 nm for all lipid compositions. (**B**) Liposomes prepared using the 100-nm filter. The measurements were done for 60, 70 and 100 % DOPC (the remaining part was DOPG, except for the 70% DOPC sample where the remaining part was CL). The mean diameter was 90±25 nm for all liposomes. DOPC vesicles with 30 % DOPE were found to be larger with a mean diameter of 120±15 nm.





**Figure S2**. Changes in the luminescence of the luciferase-luciferin couple as a result of ATP synthesis driven by an electrochemical gradient (see also **Figure 2A** in the manuscript).

Figure S3. Passive proton permeability of liposomes.

**(A)** ACMA fluorescence change for 100 % DOPC (black) and 60:40 % DOPC:DOPG (red) over time after addition of liposomes (inside pH 6) to a solution containing HEPES buffer at pH 8, with ACMA present. Samples were added at t = 0.5 min. **(B)** Changes in the fluorescence ratio of pyranine measured at 510 nm (excited at 406 nm divided by that excited at 460 nm) as a function of time after addition of HCl at t=0.5 min to induce a pH gradient of 1 unit across the membrane (from pH 7.5 to 6.5). The fluorescence ratio reflects changes in pH. Pyranine was present on the inside of the liposomes. The quencher (DPX) was added to the outside of the liposomes before measurements to eliminate signals from traces of Pyranine that may have remained on the outside of the liposomes. The three upper and lower graphs were measured with empty liposomes and with liposomes containing reconstituted ATP synthase, respectively.
